# A new challenge for crossbite treatment

**DOI:** 10.3389/froh.2023.1235134

**Published:** 2023-07-06

**Authors:** Vanda Urzal, Tainá Iunes, Afonso Pinhão-Ferreira

**Affiliations:** ^1^ Department of Orthodontics, Faculty of Health Sciences, Fernando Pessoa University, Porto, Portugal; ^2^ Department of Orthodontics, Faculty of Dental Medicine, University of Porto, Porto, Portugal

**Keywords:** malocclusion, crossbite, early diagnosis, elastic chains, orthodontics

## 1. Introduction

Crossbite is an alteration of the interarch relationship (1) that can occur in the anterior, posterior, or both regions (2). Worldwide, the prevalence of malocclusion is 56% (3), being 10% of crossbite in primary dentition, 11% in mixed dentition, and 5% in permanent dentition. Etiology is multifactorial (4) and may be related to heredity, oral breathing, sucking habits, and factors of occlusal kinetic origin (e.g., interference caused by deciduous canines) among others (5, 6).

The early diagnosis of this anomaly is essential to reduce the risk of dental, alveolar, and skeletal malocclusions. According to the severity of the established malocclusion, it may also present an aesthetic issue with psychosocial impact (7). The age at which the therapy is performed has psychological effects on the patient (8) and the occurrence of crossbite in primary dentition promote altered craniofacial growth with deleterious consequences that are proportional to the child's chronological age (9).

In pediatric patients, this orthodontic treatment is controversial. For some clinicians, crossbite resolution in the primary dentition will occur through physiological self-correction based on the growth process, although for others the treatment should be done in mixed dentition or when almost all permanent teeth are erupted (7, 10). Furthermore, in specific cases of crossbite intervention in primary dentition may also prevent an increasing abnormal skeletal growth that could lead to functional and aesthetic disorders (11).

This study aims to present a new treatment technique for children with crossbite, with the goal of reducing possible severe craniofacial treatments in the future.

## 2. Orthodontic technique with elastic chain

**Figure 1:**
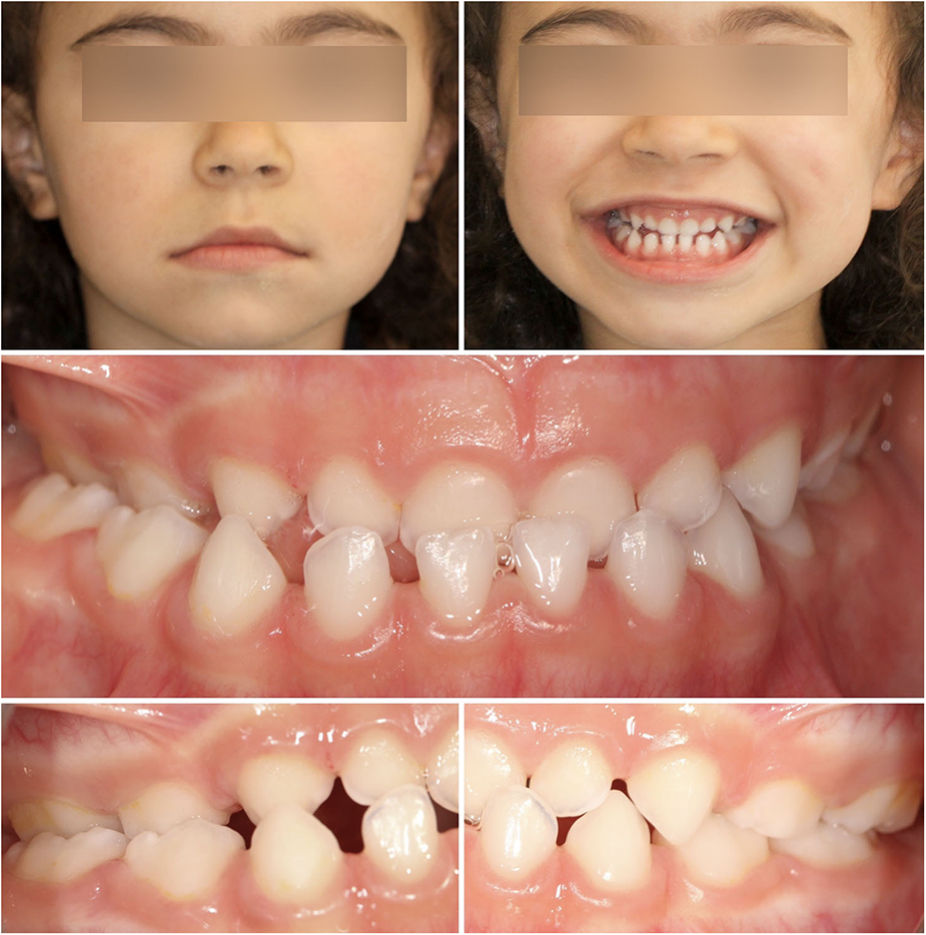
Pre-treatment of extraoral and intraoral photographs.

Buttons and elastic chains are applying in a four-year-old child patient with an anterior and unilateral crossbite of the dental arches, whose etiological factor of such dysmorphia was the lateral deviation of the mandible in the kinesics of closure (Figure 1).

**Figure 2:**
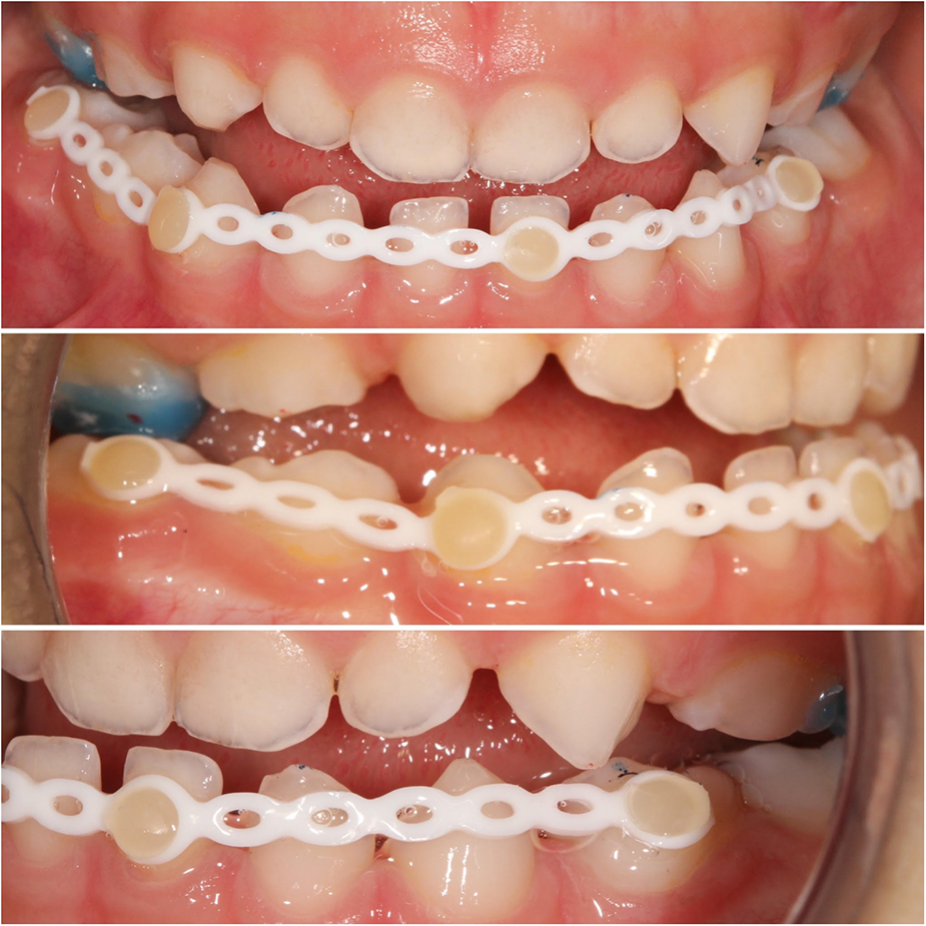
Intraoral photographs during treatment.

The treatment was carried out using four attachments to dental surface and an elastic chain. Control of the device was done carefully at short periods of time (Figure 2).

**Figure 3:**
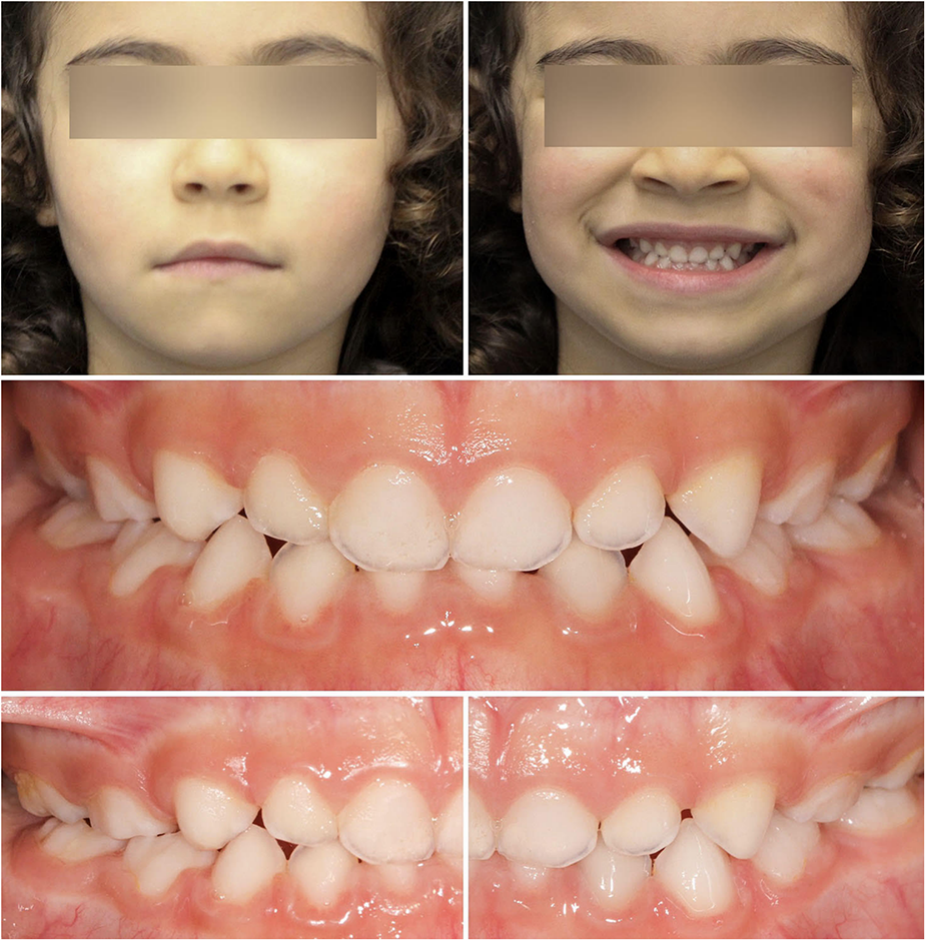
Post-treatment extraoral and intraoral photographs.

With this simple device, the correction of this malocclusion was achieved in four months, improving the dental inter-arch relationship, and promoting bilateral simultaneous skeletal growth (Figure 3). This technique has abolished the inconvenience of using a removable orthodontic appliance at this very tender age when children are not aware of treatment and the compliance is reduced.

## 3. Discussion

Elastic chains introduced in the 1960s, composed of polyurethane, are used to close or prevent spaces (12) and have such advantages as easy use, a decrease in intraoral trauma, a large range of different colors, a cost-effectiveness and a low compliance required from patients. Their behavior has been studied in many aspects: force decay over time (13–16), force decay at different levels of activation (13, 17), simulated space closure (15, 16, 18), pre stretching of elastic chains (13, 19) (causing 5% less force decrease), environmental factors and storage media (14, 19, 20) and the chains designs (13, 18).

Over time the elastic chains decrease their strength, so selecting the best quality is important. The force applied in orthodontics is complex (21) and is related to several factors: individual tissue reaction, type of applied force, and biomechanical principles involved. In any research the force, besides being measured, should be very light at its beginning, to avoid areas of hyalinization in the periodontal fibers on the pressure side. The ideal start force should be 40 gf in young people and 25 gf in adults (22). There are three types of forces: continuous, interrupted continuous, and intermittent. This is directly related to the reaction of the tissues and mechanical principles involved: a tooth with a short root will be tipped considerably and the supporting tissues will respond more favorably to interrupted or intermittent forces than to a continuous one (22). The adult tooth is tipped less than the young one due to the apical fiber bundle, and in this case, light continuous forces must be applied. In both cases, the force applied per square millimeter is higher in tipping compared to bodily movement.

Early treatment is effective and desirable in certain situations, with the clinician deciding, on a case-by-case analysis, when to perform it (23). The association of aesthetic buttons applied to the teeth with an elastic chain, and the application of a very light force, in other words, gentle stretching of the initial length of the rings, is a challenge for the possibility of earlier treatment, with less psychological interference, avoiding the patient's cooperation and preventing asymmetrical facial growth. Thus, earliest the treatment is delivered, short is the treatment length (10, 24). In the primary dentition, therapeutic options are a removable appliance and/or a McNamara disjunctor (orthopedic appliance) according to the severity and the dental age (25–27).

This simple appliance, requires excellent skills and expertise to control the movement with elastic chains in the deciduous dentition. Nevertheless, it is very important because it has solved the problem of malocclusion treatment without requiring the patient's collaboration. According to recent research, occlusion stability and adaptation to the new function are only achieved six months after conclusion and require regular assessments (28).

A technique was delivered to a four-year-old child with crossbite, whose etiological factor of such dysmorphia was the lateral deviation of the mandible in the kinesics of closure. With this simple device, the correction of this malocclusion was achieved in a short period, i.e., four months, improving the interarch relationship and promoting bilateral simultaneous skeletal growth (Figures 1, 2). However, it is important to follow up after this treatment has been concluded. Through interceptive orthodontics the malocclusion was corrected allowing normal dental, alveolar and skeletal growth, and development (29).

## 4. Conclusion

This study presents a simple technique that uses aesthetic buttons and elastic chains, with the aim of correcting the crossbite with great aesthetics and a short treatment time at earlier age.
